# Repression of Cardiac Hypertrophy by KLF15: Underlying Mechanisms and Therapeutic Implications

**DOI:** 10.1371/journal.pone.0036754

**Published:** 2012-05-07

**Authors:** Joost J. Leenders, Wino J. Wijnen, Ingeborg van der Made, Monika Hiller, Melissa Swinnen, Thierry Vandendriessche, Marinee Chuah, Yigal M. Pinto, Esther E. Creemers

**Affiliations:** 1 Heart Failure Research Center, AMC, University of Amsterdam, Amsterdam, The Netherlands; 2 Center of Molecular & Vascular Biology, University of Leuven, Leuven, Belgium; 3 Department of Gene Therapy & Regenerative Medicine, Free University of Brussels, Brussels, Belgium; Cardiovascular Research Institute Maastricht, - Maastricht University, The Netherlands

## Abstract

The Kruppel-like factor (KLF) family of transcription factors regulates diverse cell biological processes including proliferation, differentiation, survival and growth. Previous studies have shown that KLF15 inhibits cardiac hypertrophy by repressing the activity of pivotal cardiac transcription factors such as GATA4, MEF2 and myocardin. We set out this study to characterize the interaction of KLF15 with putative other transcription factors. We first show that KLF15 interacts with myocardin-related transcription factors (MRTFs) and strongly represses the transcriptional activity of MRTF-A and MRTF-B. Second, we identified a region within the C-terminal zinc fingers of KLF15 that contains the nuclear localization signal. Third, we investigated whether overexpression of KLF15 in the heart would have therapeutic potential. Using recombinant adeno-associated viruses (rAAV) we have overexpressed KLF15 specifically in the mouse heart and provide the first evidence that elevation of cardiac KLF15 levels prevents the development of cardiac hypertrophy in a model of Angiotensin II induced hypertrophy.

## Introduction

Cardiac hypertrophy is an early hallmark and important risk factor for the development of heart failure. Hypertrophy of cardiomyocytes occurs in response to pathological stimuli such as hypertension, aortic valve stenosis, myocardial infarction or genetic mutations in sarcomeric proteins and is regarded as a maladaptive process, since left ventricular hypertrophy (LVH) often progresses to heart failure [1]. Cardiac hypertrophy is accompanied by reprogramming of cardiac gene expression and the activation of ‘fetal’ cardiac genes encoding proteins involved in contraction, calcium handling and metabolism. Specific transcription factors such as MEF2, GATA4, NFAT, SRF and myocardin have been identified that activate this fetal gene program [2], [3]. Besides these transcriptional factors that promote cardiac hypertrophy, it has also become increasingly evident that the heart possesses a variety of endogenous feedback mechanisms to counterbalance this growth response [4]. We and others recently identified the transcriptional regulator KLF15 as an inhibitor of cardiac gene expression and hypertrophy [5], [6], [7]. Mouse studies showed that somatic loss of KLF15 results in increased susceptibility to pressure overload-induced LVH and heart failure [6]. The expression of KLF15 is consistently down-regulated during pathological hypertrophy and heart failure as was shown in animal models and patients with aortic stenosis and non-ischemic cardiomyopathy [5], [6], [7]. Loss of KLF15 in the heart seems unique for pathological hypertrophy, as KLF15 levels were not changed in physiological hypertrophy in rats, after exercise training [7]. The fact that KLF15 is consistently down-regulated in pathological hypertrophy may suggest that KLF15 is not just a response to heart failure, but that loss of KLF15 may actually contribute to the progression of heart failure by removing transcriptional repressive mechanisms and enabling cardiac growth [5], [6], [7], [8]. In this regard, it is remarkable that KLF15 is not (or at very low levels) expressed in the developing heart, and that its expression gradually increases after birth to reach adult levels around three weeks of age [8]. Overall, these studies indicate that overexpression of KLF15 in the heart may provide a novel therapeutic target to counteract cardiac hypertrophy and overt heart failure.

Mechanistically, KLF15 acts as a repressor of the cardiac transcription factors MEF2 and GATA4 and the transcriptional coactivator myocardin [5]. Association of KLF15 with the basic region of myocardin has been shown to prevent binding of myocardin to SRF, resulting in reduced expression of CArG box-dependent genes like ANF and α-skeletal actin [7]. Two of the family member of myocardin, Myocardin Related Transcription Factors A and B (MRTF-A and MRTF-B) also activate SRF-dependent transcription, to regulate expression of genes involved in cytoskeletal organization and muscle cell differentiation. In addition, a recent report shows that besides MYOCD, also MRTF-A is involved in the hypertrophic response of the heart [9]. It is currently unknown whether the activity of MRTF-A and MRTF-B is also repressed by KLF15. Besides repression of MEF2, GATA and myocardin by KLF15, KLF15 has also been shown to repress p300 acetyltransferase activity [6]. The observation that p300 activity is increased in KLF15 null hearts and the recognition that p300 also acetylates GATA, MEF2 and myocardin, suggest that the exaggerated hypertrophic response in the KLF15 null mice is the consequence of unbridled activity of these cardiac transcription factors [6], [10].

**Figure 1:**
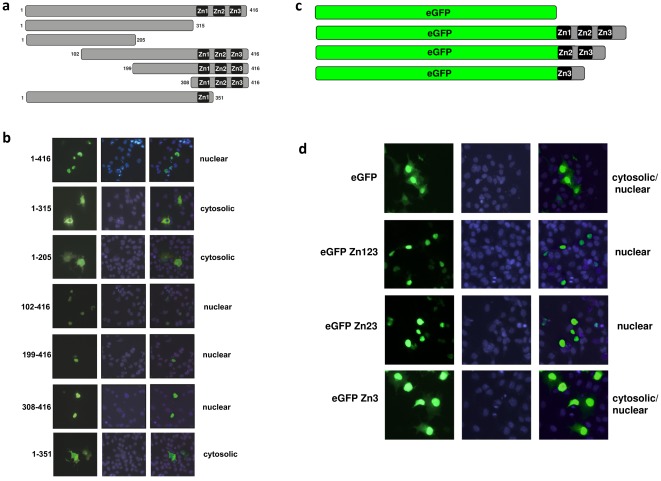
Cellular distribution of full-length and truncated KLF15 proteins. To determine which part of the KLF15 protein contains the nuclear localization signal (NLS) several truncated KLF15 open reading frames were cloned into an expression vector containing an N-terminal FLAG tag and transfected into COS-7 cells. (**a**) A schematic overview of the KLF15 truncated proteins. Full length murine KLF15 has a length of 416 aa and contains three highly conserved zinc fingers (Zn1, Zn2 and Zn3) that are located at the C-terminal end of the protein (aa315-aa416). (**b**) COS-7 cells transfected with the KLF15 expression plasmids were fixed and immunostainings were performed using a primary antibody against the FLAG-tag and a fluorescent secondary antibody (Alexa 488). Nuclei were stained with DAPI. Full length KLF15 (1–416 aa) is localized in the nucleus. Truncated KLF15 proteins lacking the NLS (1–205 aa and 1–315 aa) fail to translocate to the nucleus. All KLF15 mutant proteins lacking N-terminal parts, but containing the three zinc fingers (102–416 aa, 199–416 aa, 308–416 aa) are located in the nucleus. A KLF15 mutant lacking Zn2 and Zn3 is not located in the nucleus indicating that both Zn2 and Zn3 are necessary for nuclear localization of KLF15. (**c**) To study whether the three zinc fingers of KLF15 can acts as a NLS we fused one or more zinc fingers to eGFP. A schematic representation of the four eGFP constructs that were used are shown. (**d**) eGFP is both localized in the cytosol and the nucleus. When all three zinc fingers are fused to eGFP, eGFP is restricted to the nucleus, indicating that the three zinc fingers are sufficient to drive nuclear localization. When eGFP is only fused to Zn2 and Zn3, expression is still nuclear, but when fused to Zn3, expression is both cytosolic and nuclear, indicating that Zn2 and Zn3 act as NLS.

**Figure 2:**
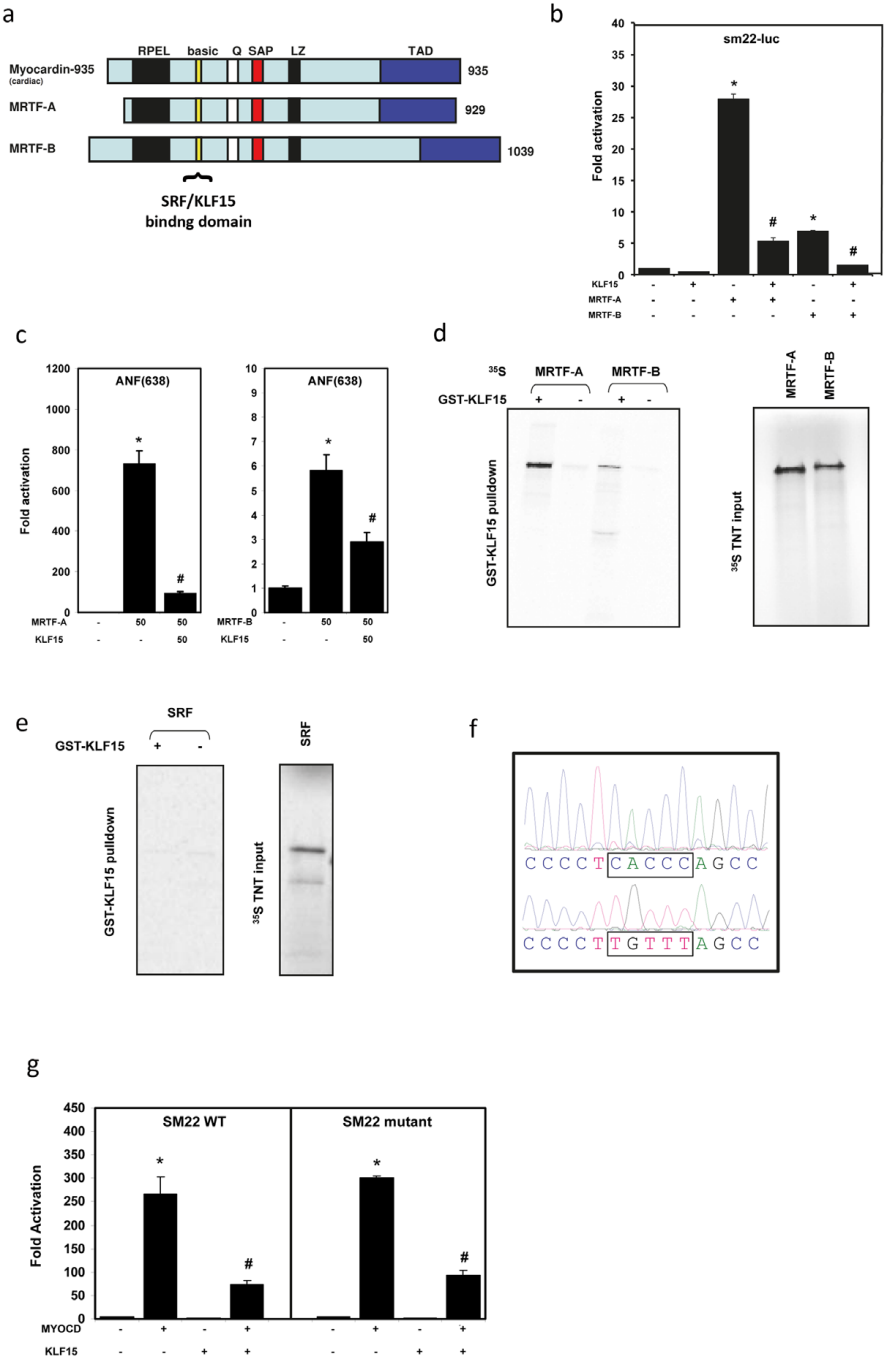
KLF15 represses and binds MYOCD and MRTF-A and –B. (**a**) schematic overview of MYOCD, MRTF-A and MRTF-B. All the proteins share a SRF binding and a (potential) KLF15 binding domain. (**b**) KLF15 represses MRTF-A and MRTF-B mediated activation of the SRF responsive −505 Sm22 luciferase reporter. (**c**) KLF15 represses MRTF-A and MRTF-B mediated activation of the SRF responsive −638 ANF luciferase reporter. (**d**) A GST-pulldown assay using in vitro translated ^35^S labeled MRTF-A and MRTF-B and GST-fused KLF15 shows a direct interaction between KLF15 and MRTF-A and –B. (**e**) GST pulldown assays using ^35^S labeled SRF and GST fused KLF15 shows no interaction between KLF15 and SRF. (**f**) KLF binding site (CACCC) in the −505 Sm22 reporter. This bindingsite is mutated to a TGTTT site. (**g**) deletion of the KLF binding site in the −505 Sm22 reporter does not affect the repressive effect of KLF15.

**Figure 3:**
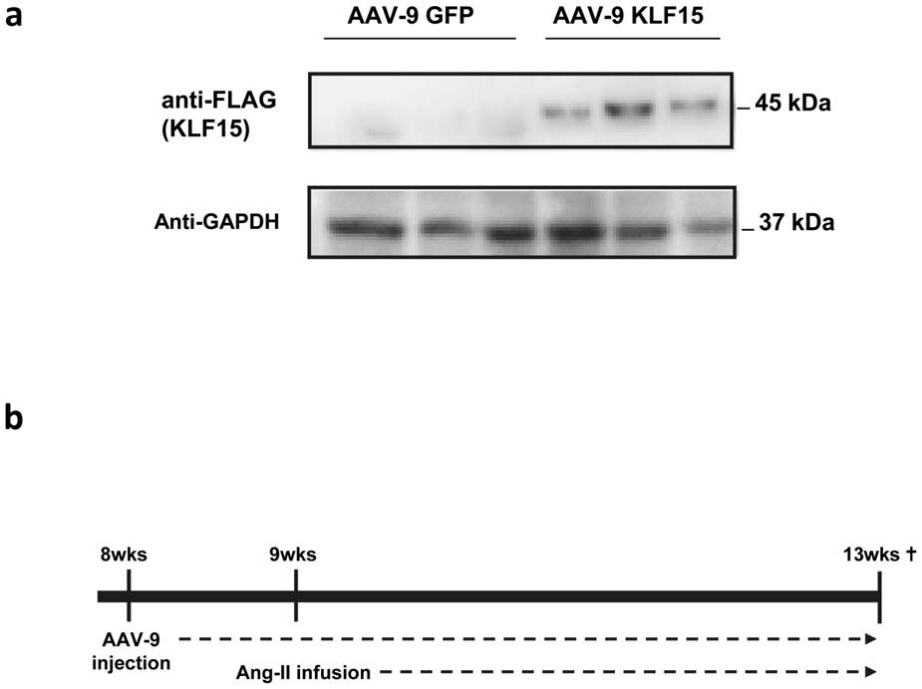
In vivo overexpression of FLAG-KLF15 using AAV9. **a**) Western blot analysis of left ventricular samples revealed expression of FLAG-KLF15 protein. **b**) Experimental timeline. Eight week old mice are intravenously injected with 1*10^10^ vector genomic copies AAV-9 (KLF or GFP). One week later hypertrophy is induced by implanting osmotic minipumps that release 1.5 µg/g/day of angiotensin II. Four weeks later, mice were sacrificed.

**Figure 4:**
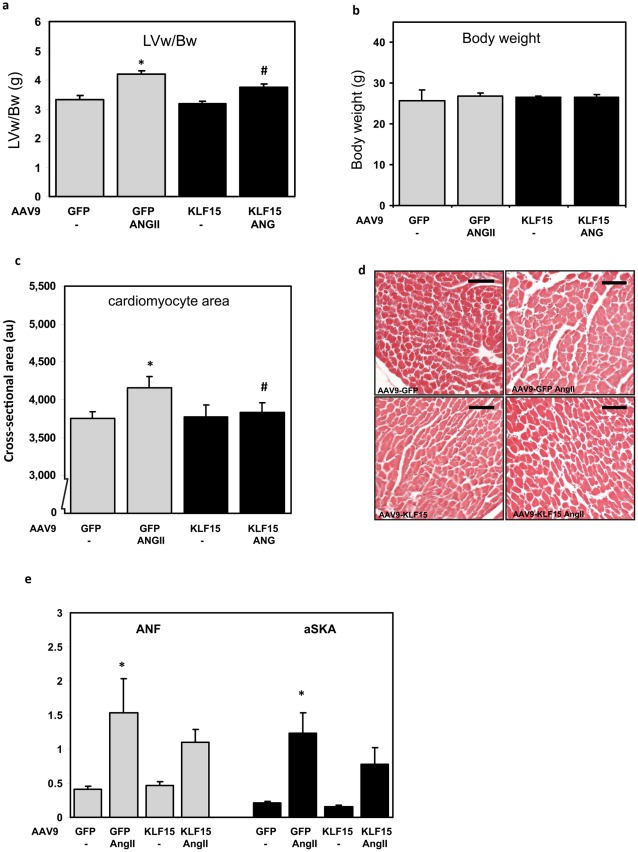
Attenuation of cardiac hypertrophy in AAV9-KLF15 mice. **a**) Body weight is not different in sham and AngII treated animals. **b**) AngII induces hypertrophy in GFP expressing mice, as measured by correcting the left ventricular weight (LVw) for body weight (Bw). This effect is blunted in mice with cardiac specific overexpression of KLF15. **c and d**) The hypertrophic response was also measured by analysis of individual cardiomyocyte size. Mice overexpressing KLF15 showed a blunted hypertrophic response to AngII. AU: arbitrary units. Scale bar in d represents 50 µm **e**) Quantitative real-time PCR on left ventricular tissue showed an increased expression of the hypertrophic markers ANF and alpha skeletal actin (aSKA) in GFP mice treated with angiotensin. In mice that overexpress KLF15 we found a reduction in expression of these genes, but this is not significant. * p<0.05 compared to GFP control. # p<0.05 compared to GFP ANGII.

**Figure 5:**
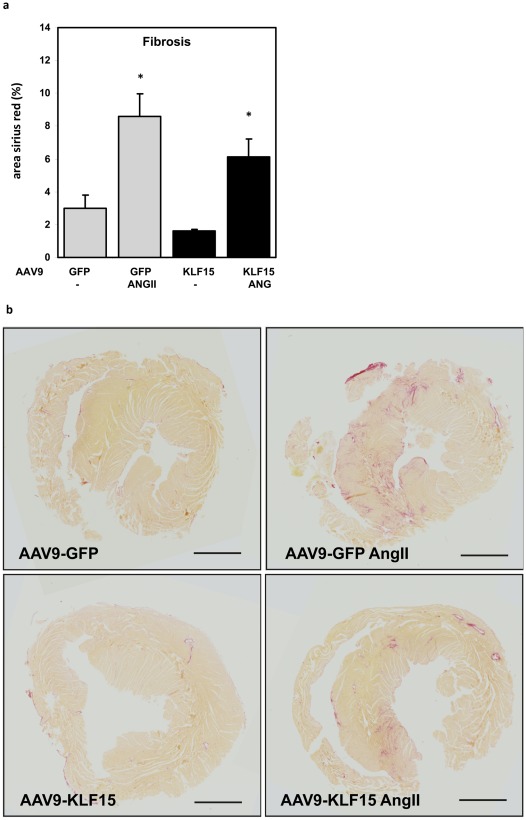
Fibrosis is attenuated by KLF15 overexpression. **a**) Upon induction of hypertrophy, fibrosis is increased in GFP mice. In mice overexpressing KLF15, fibrosis seems reduced but the difference did not reach statistical significancy. **b**) Sirius red sections. *p<0.05 compared to GFP control. Black scale bar represents 2 mm.

Despite growing evidence for a role of KLF15 as a regulator of many biological and pathological processes, only few studies have addressed the function of the different domains within the KLF15 protein [11], [12]. The only functional domain of KLF15 identified thus far is the zinc finger region at the C-terminus, which is required for DNA interaction [11], [13].

We set out this study to characterize the interaction of KLF15 with MRTFs and found that KLF15 strongly represses the transcriptional activity of MRTF-A and MRTF-B. Second, we identified a region within KLF15 that contains the nuclear localization signal. Third, we investigated if overexpression of KLF15 in the heart has therapeutic potential for the repression of cardiac hypertrophy. Using recombinant adeno-associated virusses (rAAV) we have overexpressed KLF15 specifically in the mouse heart and provide the first evidence that elevation of cardiac KLF15 levels prevents the development of cardiac hypertrophy in a model of Angiotensin II induced hypertrophy.

## Materials and Methods

### Plasmids

The ANF(638) and the SM22(505) luciferase reporter plasmids were previously described [7]. MYOCD, MRTF-A and -B pcDNA3.1 expression vectors were kindly provided by Dr. E. N Olson (Dallas, USA). The KLF15 pcDNA3.1 N-FLAG expression and PGEX-GST-wtKLF15 vector were described previously [7]. All KLF15 mutants were generated by PCR using the KLF15 pcDNA3.1 plasmid as a template. GFP-zinc finger fusion plasmids were generated by cloning the GFP open reading frame lacking the stop codon in the pcDNA3.1 vector upstream of the zinc fingers. All plasmids were sequence verified and all primer sequences are available upon request.

### Luciferase assays

Luciferase assays were performed as previously described [7]. In short, 75 ng of reporter plasmid, 50 ng of each expression plasmid (unless otherwise indicated) and 30 ng of beta-galactosidase were transfected in a 24 well plate seeded with 50.000 COS-7 cells per well using GeneJammer as a transfectant (Stratagene). After 48 hours, cells were harvested and luciferase signals were measured using a Glomax plate reader. Experiments were performed in duplicate and repeated at least three times.

### GST pulldown

GST-KLF15 and GST-empty plasmids were transformed into Bl21-Gold (DE3) cells (Stratagene) and protein translation was induced by adding 0.5 mM Isopropyl β-D-1-thiogalactopyranoside (IPTG). Proteins were isolated and bound to Glutathione Sepharose 4B Beads (GE Health Care, 17-0756-01). Quality of the GST proteins was checked by running 5 ul of protein on a 10% SDS gel followed by staining with PageBlue™ protein staining solution (Fermentas). All ^35^S labelled proteins were translated using a cell free TNT quick coupled translation system (Promega, L1170) and labelled with ^35^S methionine (Perkin Elmer). Proteins labelled with ^35^S were assayed for binding to GST-KLF15.

### Cytochemical staining

To determine cellular distribution of the KLF15 mutants, COS-7 cells were seeded in 24-well plates and transfected with 500 ng expression plasmid using GeneJammer (Stratagene). Twenty-four hours after transfection, cells were washed with PBS and fixed with 4% paraformaldehyde for 15 minutes at room temperature. Cells were washed with PBS and permeabilized for 10 minutes with 0.1% triton-X in PBS. After two PBS washes, cells were incubated at 37°C for 45 minutes with a primary antibody against the FLAG epitope (1∶250) using the anti-FLAG-M2 antibody (Stratagene). Cells were washed with PBS and exposed at 37°C for 45 min with an Alexa532-conjugated goat-anti mouse antibody (1∶200) (# A-11017, Invitrogen, Molecular probes). Nuclei were stained with DAPI.

### Animal studies and AAV9 construction

The described studies are comprised within gene transfer/therapy research of the University of Leuven, which was reviewed and approved by the university Ethical Commission for Animal Experimentation (file N° P03102). Precautions were taken to minimize animal suffering by implementing anesthesia (e.g. isofluorane) for implantation of osmotic minipumps and to perform to euthanasia by cervical dislocation when the experiment was terminated.

pAAV9-cTnT-GFP and pAAV9-cTnT-FLAG-KLF15 were derived by subcloning the open reading frame of GFP and mouse KLF15 into pAAV-MCS (Stratagene). Subsequently, the CMV promoter was removed by restriction and replaced by the −374+38 chicken troponin T promoter. Virus was produced in HEK293 cells and purified as previously described [14], [15]. 10 e^10^ vector genomic copies (vg) of AAV9-KLF15 and AAV9-GFP were injected into the tail vein of eight week old male C57BL/6 mice (n = 4 in the control groups and n = 5 in the AngII groups). After one week, mini-pumps (ALZET model 2004; ALZACorp., Palo Alto, California, USA) filled with either AngII (H-1705, Bachem) or saline and a pumping rate of 1.5 ug/g/day were placed subcutaneously as described earlier [16]. After 28 days, mice were sacrificed under 2.5% isofluorane. KLF15-FLAG protein levels were measured by Western Blotting using an anti-FLAG M2 antibody 1∶500 (Stratagene) and anti-GAPDH 1∶5000 (Fitzgerald Industries International).

### Histochemical Studies

Mouse hearts were fixed overnight in 4% paraformaldehyde, dehydrated, and embedded in paraffin using standard techniques. Consecutive 7-µm serial sections were cut and stained with hematoxylin and azophloxine. To determine the collagen content, deparaffinized sections were processed for Picrosirius red stain.

### Cell size measurements

Cell size was determined in hematoxylin and azophloxine- stained sections using 20× magnification. To determine cell size, we measured cross-sectional areas of ∼150 individual transversely cut cardiomyocytes using the image processing software Scion Image. Cross-sectional areas are expressed as arbitrary units (AU).

### Sirius Red quantification

Sirius red staining was quantified by using an in house made quantification macro. Per heart/section, 160 fields were recorded and of those 160 fields, 20 fields were randomly chosen for quantification. Perivascular fibrosis was manually omitted from the sections. The percentage of Sirius red positive areas in each section was automatically calculated as a percentage of the total tissue area.

### Statistical Analysis

Data are presented as mean ± standard errors. Unpaired t test was used to calculate p-values. P values of ≤0.05 were considered statistically significant.

## Results

### The three zinc fingers of KLF15 are required for nuclear localization

One requirement for a transcription factor to function is that it has to be translocated to the nucleus. Since KLF15 lacks the typical nuclear localization signal (NLS) PKKKRKV, KLF15 must have an alternative sequence to drive nuclear import. To identify the region that contains the NLS of KLF15 we subcloned several flag-tagged deletion mutants of KLF15 and overexpressed these in COS7 cells. We performed immunostainings with an antibody against the flag epitope to determine its cellular localization. In **Figure 1a** a schematic representation can be appreciated of the deletion mutants of KLF15 that we generated to identify the NLS. As can be appreciated from **Figure 1b**, mutants 1–205 aa and 1–315 aa, both lacking the three zinc fingers (Zn1, Zn2 and Zn2), showed solely a cytosolic expression pattern, which indicates that the C-terminal zinc finger region contains the NLS. Indeed, all KLF15 mutants containing the three zinc fingers were correctly and fully localized in the nucleus. To further narrow down this region we used a mutant in which we deleted the two most C-terminal zinc fingers (1–351 aa). The protein generated from this mutant was expressed in both the nucleus and cytosol, indicating that the region encompassing the two most C-terminal zinc fingers is required for complete nuclear localization (**Figure 1b**).

To study whether the zinc fingers are not only necessary for nuclear localization but also sufficient to act as an NLS, we created an expression vector containing the open reading frame of enhanced green fluorescent protein (eGFP) fused to one or more zinc fingers of KLF15 (**Figure 1c**). We transfected these plasmids in COS-7 cells and the cellular localization was determined using fluorescence microscopy. Naive eGFP is primarily localized in the cytoplasm, but since it is a relatively small protein it easily translocates to the nucleus by unspecific diffussion [17]. Fusion of all three zinc fingers to the C-terminal end of eGFP (eGFP-Zn123) resulted in an expression pattern that is completely restricted to the nucleus (**Figure 1d**), indicating that the zinc finger region is sufficient to act as an NLS. eGFP fused to Zn2 and Zn3 also showed a nuclear expression whereas fusion of Zn3 only resulted in both cytosolic and nuclear expression (**Figure 2d**). From these two experiments we conclude that zinc fingers 2 and 3 are required to translocate KLF15 to the nucleus.

### KLF15 represses the activity of MRTF-A and B

We have previously shown that KLF15 physically interacts with MYOCD resulting in a strong repression of MYOCD activity [7]. Here, we hypothesize that KLF15 represses the activity of other SRF co-activators like MRTF-A and MRTF-B. These proteins are family members of MYOCD and essentially contain the same functional domains (**Figure 2a**) [18]. To test whether KLF15 is able to repress the activity of MRTF-A and -B, we performed luciferase assays using the CArG box-containing SM22 (505) and ANF(638) reporters, which are strongly activated by myocardin, MRTF-A and –B [19]. Co-transfection of COS7 cells with these reporters, MRTF-A or -B and KLF15 showed that KLF15 strongly repressed MRTF-A and -B activity on both the SM22 and the ANF reporter (**Figure 2b and c**). Next, we tested using GST pulldown studies, whether KLF15 could directly interact with both MRTFs. We used a GST-KLF15 fusion protein as a bait to test for interaction with *in vitro* translated, ^35^S-labeled MRTF-A and –B and show that KLF15 directly interacts with both MRTFs (**Figure 2d**).

Recently, it was shown that a synergistic interaction between KLF3 and SRF controls transcription of skeletal muscle genes [20]. Interestingly, activation of SRF occurred via recruitment of SRF to KLF binding sites in the muscle creatine kinase promoter, independent from its association to CArG boxes [20]. This finding prompted us to examine whether KLF15 is able to physically interact with SRF and we performed GST pulldown assays using a GST-KLF15 fusion protein and *in vitro* translated ^35^S labeled SRF. This revealed that there is no direct interaction between SRF and KLF15 (**Figure 2d**), further underscoring that KLF15 represses SRF-mediated transcription not by binding to SRF, but through direct binding to MYOCD and MRTFs. Along the same lines, we tested whether KLF15 competes with SRF for MRTF binding by transfecting Cos cells with the Sm22-luciferase reporter and expression vectors encoding MYOCD, MRTF-A or MRTF-B. Luciferase assays revealed that the repression of MRTF-A and –B by KLF15 is dose-dependent and that this repression can be counteracted by increasing concentrations of SRF (Supplemental Figure S2).

### KLF15 represses the SM22 reporter independent of the KLF15 binding site

Since it is currently unknown whether consensus KLF binding sites in the promoters/enhancers of cardiac genes regulate gene expression, we searched for KLF binding sites in promoters of genes known to be repressed by KLF15. We noted a typical KLF binding site (CACCC) in the promoter of SM22, 122 bp upstream of the CArG box (**Suppl Figure S1a**) and several KLFs (KLF2, 3, 8 and 17) have been shown to bind this sequence [21], [22], [23]. To investigate whether this KLF binding site is required for the KLF15-mediated repression of the SM22 reporter, we mutated this binding site (CACCC to TGTTT) (**Figure 2f**) and performed luciferase assays using MYOCD to activate the wild-type (WT) and mutated reporter. Since KLF15 similarly repressed WT and mutated SM22 reporter (**Figure 2e**), we conclude that this KLF binding site in the promoter of Sm22 is not required for the repressive action of KL15. This provides further evidence that KLF15 mainly represses gene transcription through interaction with MYOCD or MRTFs.

### AAV9-mediated gene transfer of KLF15 represses cardiac hypertrophy

KLF15 is an endogenous repressor of cardiac hypertrophy, as was evidenced by the fact that KLF15 null mice have exaggerated cardiac hypertrophy and develop heart failure [5], [6]. The fact that KLF15 is consistently down-regulated in animal models of hypertrophy and human heart failure may suggest that KLF15 is not a just a response to heart failure, but that loss of KLF15 may actually contribute in the disease progression [5], [6], [7], [8]. This prompted us to investigate whether overexpression of KLF15 in the heart, sufficient to prevent its down-regulation, would be able to repress the development of hypertrophy, in a model of AngII-induced LVH.

To increase cardiac levels of KLF15 we designed rAAV9 for overexpression of KLF15 under the control of the −374 chicken cardiac troponin T promoter. This promoter has been shown to efficiently drive the expression of SERCA in cultured rat cardiomyocytes [24], [25]. To generate a rAAV9-KLF15 construct that is only active in the heart we subcloned the chicken cTNT promoter upstream of FLAG-tagged KLF15 open reading frame. As a control, we generated a cTnT-eGFP construct. Both sequences were subcloned into a rAAV-9 vector and virus was produced as previously described [15]. To establish AAV-9 mediated gene transfer, eight week old male C57Bl6 mice were intravenously (i.v.) injected with 10 e^10^ vector genomic copies of rAAV9-KLF15 and rAAV9-GFP. Five weeks after injection we assessed whether we could detect FLAG-tagged KLF15 in the hearts of animals that were transduced with AAV9-KLF15. Western blot analysis on cardiac tissue lysates with an antibody directed against the FLAG epitope revealed that recombinant KLF15 protein was efficiently produced in the hearts of AAV9-KLF15 injected mice (**Figure 3a**).

To determine whether overexpression of KLF15 represses pressure overload-induced cardiac hypertrophy, we induced hypertrophy a week after injecting mice with the rAAV9-KLF15 and rAAV9–GFP virus, through continuous infusion of AngII (1.5 µg/g/day) via subcutaneously placed osmotic minipumps (**Figure 3b**) [16]. As can be appreciated from **Figure 4a**, rAAV9-GFP infected mice develop a mild but statistically significant increase of 25% in left ventricular weight (LVw) when corrected for body weight (Bw) after 4 weeks of AngII infusion. The body weights between groups are not significantly different (**Figure 4b**). AngII treatment of the rAAV9-KLF15 mice resulted in a blunted increase in LVw/Bw ratio as compared to the mice infected with AAV9-GFP. This indicates that forced overexpression of KLF15 can inhibit the hypertrophic response of the heart.

Next, we assessed whether KLF15 overexpression affects hypertrophic growth of individual cardiomyocytes. We measured cross-sectional areas of ∼150 individual cardiomyocytes in LV tissue on H&E stained sections. As can be appreciated from **Figure 4c and d**, the cross-sectional area of cardiomyoctyes in the control group (rAAV9-GFP) is significantly increased (11%) in response to AngII. In contrast, in the rAAV9-KLF15 mice this increase in cell size was nearly abolished. We quantified the expression of established hypertrophic marker such as atrial natriuretic factor (ANF) and alpha skeletal actin (αSKA) using real-time PCR on hearts and showed that the induction of these marker genes in response to AngII treatment was slightly attenuated in the rAAV9-KLF15 treated mice. (**Figure 4e**), however this attenuation in gene expression did not reach statistical significance.

The presence of fibrosis is another characteristic of LVH and heart failure, and KLF15 has also been implicated in this process [26]. We performed sirius red stainings on cardiac sections to determine the amount of interstitial fibrosis in rAAV9-GFP and rAAV9-KLF15 mice. As can be appreciated from **Figure 5a and b** AngII infusion results in increased collagen content in AngII treated hearts. In hearts of AAV9-KLF15 mice treated with AngII, the increase in fibrosis is attenuated, but this difference did not reach statistical significance.

## Discussion

MYOCD is a transcriptional co-activator whose expression is restricted to cardiomyocytes and smooth muscle cells, where it acts as a powerful co-activator of serum response factor (SRF) and myocyte enhancer factor 2 (MEF2), two transcription factors with an established role in cardiac biology [27], [28], [29]. A recent report shows that besides MYOCD, also MRTF-A is required for the hypertrophic response of the heart [9]. In this study we show that KLF15 not only binds and represses MYOCD, but also represses the transcriptional activity of MRTF-A and MRTF-B. Although we did not pinpoint the exact KLF15 interaction domain within the MRTFs, it is likely that this region is similar to the one within MYOCD, since there is high sequence homology between the MYOCD family members, especially in the basic region (i.e. the SRF binding region, where KLF15 is also known to bind). Since MRTFs are expressed in a wide range of tissues, these findings suggest roles for KLF15 in regulating SRF-dependent transcription in other cell types and organs as well. In this regard, a recent study shows that MRTF-A controls myofibroblast activation and fibrosis in response to myocardial infarction [30]. Other reports indicate that MRTFs and SRF play critical roles in cytoskeletal dynamics and metastasis [31], smooth muscle biology [32] and skeletal muscle growth [33]. A requirement for a role for KLF15 in these processes is that KLF15 has to be expressed in these tissues. Uchida et al. and Gray et al. showed that KLF15 is ubiquitously expressed and that it is enriched in smooth, cardiac and skeletal muscle as well as in the kidney and liver [8], [34].

Although it is known that KLF15 is a repressor of several transcriptional regulators, the exact mechanism by which KLF15 represses transcriptional activity remains unknown. This is largely because of a lack of knowledge on the functional domains within the KLF15 protein. The zinc finger region at the c-terminus of KLF15 is the only functional domain described so far. The zinc fingers are required for DNA binding [13], and as we have shown in this study, also to gain entry to the nucleus. This is in agreement with several other KLFs, where the NLS has also been identified within the zinc finger regions [35].

During cardiac hypertrophy and heart failure, KLF15 is consistently downregulated in patients, mice and rat models [5], [7]. Loss of KLF15 correlates with the substantial increase in ANF and BNP expression, and the experiments in this study provide a putative model how loss of KLF15 may contribute to increased expression of ANF, BNP and α-skeletal actin, and possibly other SRF- dependent target genes. In this study, we have investigated the therapeutic potential of KLF15. We investigated whether overexpression of KLF15 in the mouse heart is sufficient to prevent the development of cardiac hypertrophy and fibrosis in response to AngII-induced hypertension. Although *in vitro* studies have previously revealed an inhibitory effect of KLF15 overexpression on cell growth, *in vivo* studies were lacking [5].

Efficient and long-term delivery of potential therapeutic genes to the heart remains a major challenge for clinical implementation. One of the most promising tools in this context are the AAVs, which are able to take up large DNA fragments and enable stable gene transfer to the heart [36]. Furthermore, rAAVs are non-pathogenic, as they are replication-deficient and do not evoke an immune response. AAV vectors have been studied for the past 30 years and these parvovirus-related viruses currently encompasses 9 serotypes [37], [38]. With the discovery of different AAV serotypes and variants it became evident that serotypes can be used to establish an organ-specific expression of the transgene [39]. Two recent studies showed that specifically the AAV9 serotype efficiently transduces the heart after intravenous delivery [40], [41]. This is in contrast to rAAV-1, rAAV-2 and rAAV-6 which only slightly transduce the heart when high doses of viral genome are injected. rAAV-8 does efficiently transduce cardiac tissue, but relatively high virus doses are needed [36].

Swinnen et al. showed that rAAV9-mediated gene transfer is not only very efficient, but it also has therapeutic potential for heart disease. When they overexpressed thrombospondin-2 (TSP-2) using AAV9-CMV-TSP vectors in the hearts of TSP-2 null mice they were able to rescue accelerated age-induced cardiomyopathy completely [15]. In 2009, Jaski et al. performed a human phase 1/2 clinical trial using rAAV1 to overexpress SERCA2a in 9 patients with advanced heart failure [42]. This study did not raise any safety concerns. Even though the study cohort was too small to conduct statistical analysis, some patients showed improvements in LV function (ejection fraction and end systolic volume), biomarkers (NT-proBNP) and function (walking test and VO2 max). The above described findings emphasize that AAV mediated gene therapy is a potentially efficient and successful way to overexpress therapeutic targets, however further clinical evaluation is warranted.

Specificity of gene transfer to cardiomyocytes can be increased by incorporating a cardiac promoter, instead of the CMV promoter into the AAV9 vector [37], [43]. In the current study, but also in the study of Prasad et al., the chicken cardiac troponin T (cTnT) −374+43 promoter was introduced in the rAAV9 vector [36]. This promoter has been shown to efficiently drive transgene expression in cardiomyocytes [24], [25] and Prasad et al. provided evidence that the cTnT-rAAV9-eGFP vector efficiently transduces the heart (up to 96% of cardiac cells) resulting in high eGFP expression levels. In the current study, we have used western blot analysis to demonstrate that cTnT-rAAV9-KLF15 transduction results in the generation of KLF15 protein in the heart.

Previous work of Wang et al. showed that KLF15 also has a function in the cardiac fibroblast, where it represses the expression of the pro-fibrotic cytokine CTGF [16], [26] and inhibits ECM synthesis. Overall, excessive deposition of ECM is a negative hallmark of cardiac remodeling since it stiffens the heart thereby hindering proper contraction. Mice lacking KLF15 are not only sensitized to the development of hypertrophy and heart failure, but they also develop severe fibrosis [26]. In the AAV9-KLF-15 treated mice, we did not find a statistically significant inhibition on the development of fibrosis, which may be explained by the fact that we did not overexpress KLF15 in the fibroblast but in the cardiomyocyte However, since CTGF is also expressed and secreted by cardiomyocytes (especially in the stressed heart), it could very well be possible that the mechanism of CTGF repression by KLF15 is shared by the myocytes of the heart [44] [26].

In conclusion, the fact that cardiomyocyte-specific KLF15 overexpression may reduce cardiomyocyte hypertrophy seems beneficial and suggests that KLF15 may be used as a therapeutic target for hypertrophy and heart failure. Future studies are warranted to establish whether overexpression of KLF15 improves cardiac function and whether it can also prevent hypertrophy in more severe models of hypertrophy and heart failure, such as transverse aortic constriction.

## Supporting Information

Figure S1 **Sequence of the −505 sm22 and −423+98 hBNP reporters.** The Sm22 and BNP reporters contain several conserved transcription factor binding sites: CArG-box (yellow), GATA (blue), KLF (red).
(TIF)

Figure S2 **KLF15 competes with SRF for MRTF interaction.** Cos cells were transfected with Sm22 luciferase and expression vectors encoding myoardin (panel A), MRTF-A (panel B) or MRTF-B (panel C) and increasing concentrations of KLF15 and SRF as indicated. Luciferase activity is expressed as fold change over the empty expression vector pcDNA3.1. 50 indicates that 50 ng was transfected to a well of a 24-well plate. Increasing concentrations indicated by the triangle range from 1 to 50 ng.
(TIF)
