# Supplementary figures and images for: Repression of Cardiac Hypertrophy by KLF15: Underlying Mechanisms and Therapeutic Implications
Source: PLoS One. 2012 May 7;7(5):e36754. doi: 10.1371/journal.pone.0036754 (PMC3346753; doi:10.1371/journal.pone.0036754)

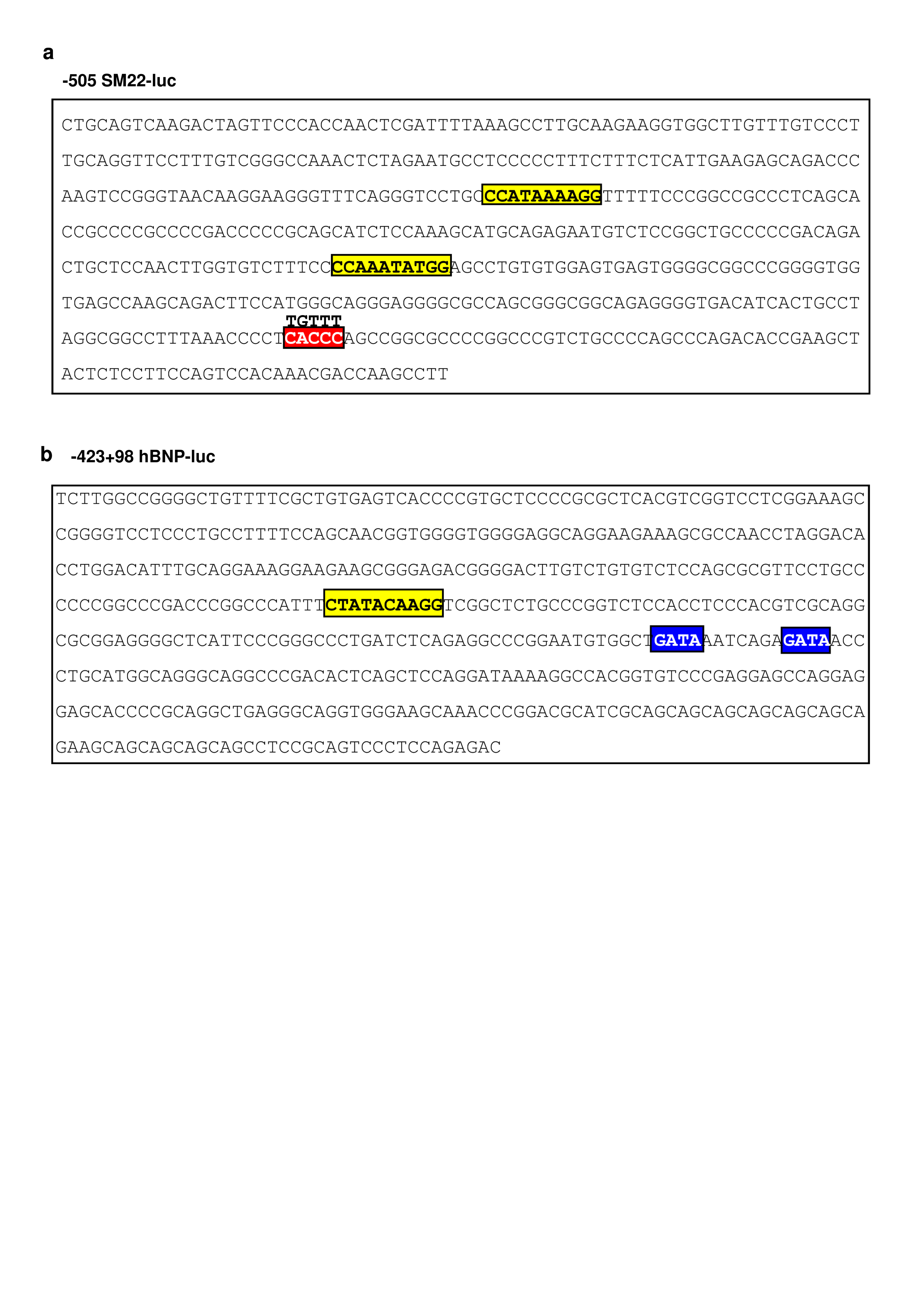

Supplement: Figure S1 — Sequence of the −505 sm22 and −423+98 hBNP reporters. The Sm22 and BNP reporters contain several conserved transcription factor binding sites: CArG-box (yellow), GATA (blue), KLF (red). (TIF) [file pone.0036754.s001.tif]
